# The Delusion of Growing Fat

**Published:** 1885-08

**Authors:** 


### The Delusion of Growing Fat.

If you continue your present dietary and habits, and live five or seven years more, the burden of fat will be doubled, and the insinuating tailor will be still congratulating you. Meantime you are "running the race of life"—a figure of speech less appropriate to you at the present moment, than it formerly was—handicapped by a weight which makes active movement difficult, upstairs ascents troublesome, respiration thick and panting.

Not one man in fifty lives to a good old age in this condition. The typical man of eighty or ninety years, still retaining a respectable amount of energy of body and mind, is lean and spare, and lives on slender rations.

Neither your heart nor your lungs can act easily and healthily, being oppressed by the gradually gathering fat around. And this is because you continue to eat and drink as you did, or even more luxuriously than you did, when youth and activity disposed of that moiety of food which was consumed over and above what the body required for sustenance.

Such is the import of that balance of unexpended aliment which your tailor and your foolish friends admire, and the gradual disappearance of which, should you recover your senses and diminish it, they will still deplore, half frightening you back to your old habits again by saying :

"You are growing thin ; what can be the matter with you?"

Insane and mischievous delusion.—*Nineteenth Century.*

